# Evaluation of Behavior and Affective State of Different-Parity Sows with Strong/Weak Pupil Light Reflex

**DOI:** 10.3390/ani12091184

**Published:** 2022-05-05

**Authors:** Jinyue Zhang, Langchao Yu, Guoan Yin

**Affiliations:** College of Animal Science and Veterinary Medicine, Heilongjiang Bayi Agricultural University, Daqing 163319, China; 18345449957@163.com (J.Z.); qaxylc@163.com (L.Y.)

**Keywords:** pupil light reflex, confined sows, stereotypic behavior, affective state

## Abstract

**Simple Summary:**

Pupil light reflex (PLR) is controlled by the sympathetic and parasympathetic nervous system and is a sensitive indicator of the affective state of animals. Pupil rigidity was observed in sows in long-term confinement, and there were differences in the PLR of sows of the same parity. This study investigated the differences in the behaviors and affective state of sows with different PLR and parity. Compared with sows in the strong PLR group (SR), those in the weak PLR group (WR) performed less standing and lateral lying, and more ventral lying and sitting behaviors. In a novel object test (NOT), the number of novel object contacts and contact duration of WR sows were less than in SR sows, and the response latency time of WR sows was longer than SR sows. High-parity sows showed anhedonia and lack of motivation. The affective state of sows changed significantly with an increase in parity. Sucrose and quinine responses also verified that sows showed more severe affective disorders in higher parity. Thus, PLR may be a potent indicator for evaluating the behaviors and affective state of sows.

**Abstract:**

The stall-housing system is commonly used in the modern swine industry in many countries; however, long-term space restrictions can cause affective and physiological abnormalities in sows. The pupil light reflex (PLR) can reflect the psychological and neurological changes in animals, and confined sows show higher pupillary rigidity. However, the PLR differs between same-parity sows, suggesting differences in behaviors and affective states between parity groups. We subjected confined Yorkshire × Landrace sows of parity 0, 2, and 5 to a PLR test and accordingly assigned them to the weak PLR (WR) group (*n* = 20) or the strong PLR (SR) group (*n* = 22). We then observed the sows’ behaviors and performed a sucrose/quinine response test and novel object test (NOT) to assess the differences in their affective states. The standing and lateral lying behaviors of the sows were less frequent in WR than in SR (*p* < 0.05), whereas ventral lying and sitting behaviors was more frequent in WR than in SR (*p* < 0.05). No changes in chewing behaviors and sucrose/quinine responses were observed (*p* > 0.05); however, the numbers and duration of novel object contact were lower and the novel object response latency time was longer in WR than in SR (*p* < 0.05). Regarding parity, standing and lateral lying behaviors were less frequent and ventral lying and sitting behaviors were more frequent at parity 5 than at parity 0 (*p* < 0.05). Bar-biting, rooting, trough-biting, and sucrose response score were lower at parity 5 than at parity 0 (*p* < 0.05), and vacuum chewing behavior and quinine response score were higher in sows of parity 5 than in those of parity 0 (*p* < 0.05). NOT showed that the number of contacts and contact duration in sows decreased with increasing parity (*p* < 0.05), and the response latency time was longer in sows of parity 5 than in those of lower parity (*p* < 0.05). In conclusion, the behavioral expression and responses of confined sows to novel objects differed between PLRs. The evaluation of the affective state of sows also revealed marked differences with increasing parity. Thus, confined sows with WR and high parity apparently suffer from more severe psychological problems, and PLR may be a potent indicator for evaluating the affective state of confined sows.

## 1. Introduction

Sows are frequently confined in crates to maximize space utilization in intensive pig farming; thus, behaviors such as turning, exploring, and learning in pregnant sows are considerably limited in restricted and barren environments [1]. The restriction of these behaviors can generate increased stress [2]. Compared with sows housed in individual stalls, sows housed in a group housing system display more exploratory behavior, less vacuum chewing, and less sitting behavior [3]. To avoid undesirable stimuli, animals may change their behaviors in terms of pattern, frequency, and intensity, compared to normal behaviors, which may result in stereotypies [4]. Stereotypic behaviors are mechanisms by which animals respond to unfavorable environmental conditions such as long-term spatial restriction [5,6]. In pregnant sows, long-term space restriction increases oral behaviors, which may develop into stereotypic behaviors such as vacuum chewing, bar-biting, and trough-biting [7].

The expression of stereotypic behaviors in confined sows increases with confinement time, and multiparous sows show more stereotypic behaviors than gilt sows [8]. Novak et al. [9] observed that mice with higher levels of stereotypy displayed a negative cognitive bias, which was influenced by the form of stereotypy. Similar results were observed in captive tufted capuchins (Cebus apella) [10]. Zhang et al. [11] found that the stereotypic behaviors of sows at parity 5 were higher than at parity 0. However, Liu [12] found no significant difference in the stereotypic behaviors of sows at parity 2 and 3. Therefore, assessing the affective state of animals based only on the frequency of stereotypic behaviors would be inadequate.

PLR characteristics are considered as sensitive indicators to assess psychological states. PLR is controlled by sympathetic and parasympathetic nerves of the autonomic nervous system [13]. Bao et al. [14] found that PLR might be a better way to monitor the psychological status of animals. Abnormal PLR characteristics indicate a disorder of the autonomic nervous system, which may be caused by an individual psychological or neurological disorder [15]. Human patients with neurological disorders, including traumatic neurological disorders, anxiety disorders, specific phobias and depression, exhibit abnormal PLR characteristics [16,17]. Compared with group-housed sows, the PLR latencies and duration of stall-housed sows were longer [14]. Additionally, the PLR of sows is affected by age [18]. Our colleague previously found that there were differences in the psychophysiological states of sows with different PLRs and the differences increased with the increase in parity [19]. Therefore, we supposed that there were differences in the behaviors and affective states of sows with strong/weak PLR characteristics and different parity.

Affective states are the subjective experiences, feelings, or emotions of animals and are considered to be an external projection of multiple subjective aspects of neurological, physiological, behavioral, and cognitive states, and consciousness [20]. Animals can experience a variety of advanced emotions and they also have negative, neutral or positive subjective experiences [21,22]. Emotional states are classified as an important component of animal welfare [23]. Abnormal emotional states not only cause the animal’s physiological state to fluctuate, but also lead to deviations in their subjective consciousness such as preferences and motivations [24]. Currently, there are two main ways to assess the affective state of animals. One way is to allow animals to control their environment and observe the choices and decisions they make, using preference and motivation tests (including aversion tests). The other is to look for signs of deprivation, frustration or distress when the animal is confined in an environment or subjected to a treatment without any means of control [24].

Testing the reward stimulus response of sows can verify the state of anhedonia or lack of motivation in individuals with affective disorder, which helps to reveal their affective disorder types. Many animals have a natural reward circuit for sweetness [25], and the response to aversive stimuli is also an important test of individual motivation control [26]. Chronic Mild Stress (CMS) rats are insensitive to sweeteners, and their consumption of sucrose solution is greatly reduced, which is considered an indicator of anhedonia [27,28]. Depressed model and alcohol-dependent model monkeys are insensitive to bitterness [29]. Swine naturally prefer sweetness over bitterness, so testing sows’ response to sweeteners can assess anhedonia, and testing sows’ response to bitterness can test motivation. Pigs naturally love sweetness and have a natural reward circuit [25,30]. Sweetener tests have been widely used in studies of reward response [31]. Reduced sucrose preference is considered to be reflective of an anhedonia-like state [32].

Animals may respond to novel stimuli with neophilic (explorative) and/or neophobic (cautious) behavior. Variations in responsiveness to environmental change within and across species is associated with cognitive, physiological, and social propensities that reflect ontogenetic variance and the natural lifestyles of a species [33,34]. Testing reactions to novel stimuli in animals has been used in fear and anxiety studies [35,36]. Compared with sows in poor environments, sows in enriched environments showed greater diversity in behavior, greater motivation to explore novel objects, less fear and lower anxiety in novel object stimuli tests. The exploring behavior of sows decreased with an increase in parity, and the number of contacts and contact duration in regard to a novel object decreased [11].

The objective of this study was to investigate the behaviors and affective states of sows with strong/weak PLR and different parity, and clarify the applicability of PLR tests for assessing the affective state of confined sows. Our results may have important implications regarding advances in psychophysiological research on sows so as to improve animal welfare.

## 2. Materials and Methods

### 2.1. Animals and Management

All procedures involving animals were approved by the Animal Ethics Committee of the Animal Science and Veterinary College of Heilongjiang Bayi Agricultural University. This study was conducted on a commercial pig farm (Heilongjiang Damuren Animal Husbandry Co., Ltd., Wuchang, China) in Heilongjiang province, northeast China. Forty-eight Yorkshire × Landrace sows with different parities were used (16 sows at parity 0, 16 at parity 2, and 16 at parity 5). Due to the failure of pupil reflex tests in 6 sows, data were only available for 42 sows. The number of sows in parity 0, 2 and 5 was 13, 13 and 16, respectively (Table 1). All sows were bred and reared under the same management conditions and were raised in the same houses during the experimental period, the temperature of the houses remained at 18–20 °C, and the humidity was 60.0–62.0%. All sows had been pregnant for 42–95 days and were housed in identical gestation crates (the crate size was 215 cm × 65 cm × 96 cm (length × width × height)). The sows were selected through strict health checks and had been subjected to standard vaccination procedures. The physical condition of the sows was consistent, especially multiparous sows. Each sow was fed with 3.0 kg of sow feed at 06:00 every day, and they had access to water ad libitum. Health inspections and manure removal were conducted daily.

### 2.2. PLR Test

The PLR characteristics were tested with a hand-held pupillometer PLR-200 (Neur Optics, Laguna Hills, CA, USA). The PLR tests were conducted from 08:00 to 09:00, and the illuminance was <250 lx. The test was conducted by Langchao Yu. Each eye of each sow was tested three times for the following parameters at an interval of > 5 min: maximum pupil diameter, minimum pupil diameter, pupil contraction rate, pupillary response latency, average contraction velocity, maximum contraction velocity, average dilation velocity, and time for 75% recovery of the initial pupil diameter. Our colleague found that there were no significant differences in the PLR data for the left and right eyes of sows. Therefore, the PLR data for the left and right eyes were regarded as duplicated data. The transform-compute variable was used for variable analysis (SPSS, Version 16.0, IBM, Armonk, NY, USA) and a comprehensive evaluation score was calculated. A comprehensive evaluation score < 0 was denoted as WR and a comprehensive evaluation score > 0 was denoted as SR [19].

### 2.3. Behavioral Observations

Monitoring equipment (Cloud SEE H-2 intelligent network camera, Shandong Zhongwei Century Technology Co., Ltd, Jinan, China) was used to video the sows and was mounted on the feed line pipe or wall in front of sows. After screening and collating, a complete 3-day behavioral video, without human interference, was selected for manual observation. The continuous scan sampling method was used to examine the video recordings. The postures and oral behaviors of the confined sows were recorded at one scan point per minute and 1440 scan points per day. Postures included standing, ventral lying, lateral lying and sitting. Oral behaviors included bar-biting, rooting, trough-biting and vacuum chewing. The categories and definitions of the behaviors are shown in Table 2 and Table 3.

### 2.4. Sucrose/Quinine Response Test

In combination with the preliminary test results, a 4% sucrose solution and 3.193 mmol/L quinine solution were selected for the response test. Confined sows were scored according to their response to an oral injection of sucrose solution and quinine solution. Sucrose (cat. No. 10021418) and quinine (cat. No. 61004234) were purchased from Sinopharm Chemical Reagent Co., Ltd., Shanghai, China. The scoring standards for the sucrose and quinine tests are shown in Table 4 and Table 5. The sucrose score was divided into 5 grades and quinine score was divided into 3 grades, with higher scores indicating more preference.

### 2.5. Novel Object Test

The sows were transferred to an empty crate for testing, and the sows were driven back to their original crates after the test was completed before testing the next sow. All tests were completed in one day. The environment was kept quiet during the test and people avoided moving around to reduce stress. After the sows were completely adapted to the new crate, a nylon rope ball was hung directly in front of the head of the sows. Their reaction to the nylon rope balls was recorded for 5 min. Each sow was tested three times with an interval of more than 30 min. In this test, the number of contacts with the novel object, response latency time and contact duration were recorded. The definitions of the parameters are shown in Table 6 [37].

### 2.6. Statistical Analyses

All data were processed by Excel 2010 (Microsoft Corporation, Redmond, WA, USA). Statistical Product and Service Solutions (SPSS, Version 16.0, IBM, Armonk, NY, USA) was used for principal component analysis. The analysis dimension reduction factor was used for factor analysis. All data were subjected to the Analyze-Descriptive Statistics-Explore for examination of normality. The results were expressed as the mean ± SD and were plotted using GraphPad Prism 8.0 software (GraphPad Software, Inc., La Jolla, CA, USA). Behavioral data were expressed as a proportion of all behaviors. Two-way analysis of variance was used for data analyses. Statistical significance is reported at *p* < 0.05.

## 3. Results

### 3.1. Behavioral Differences between Different-Parity and PLR Groups

#### 3.1.1. Posture of Confined Sows with Different Parity and Strong/Weak PLR

**Figure 1:**
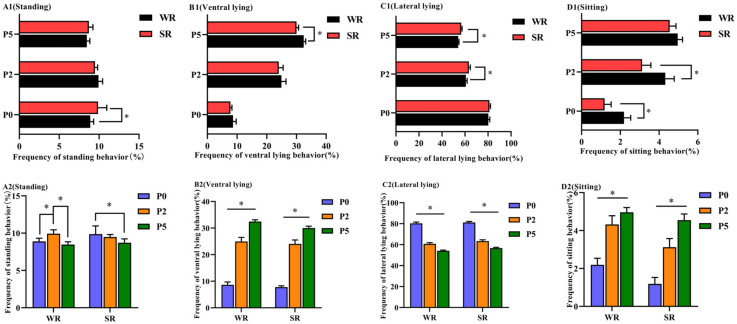
Postures of confined sows with different parity and strong/weak PLR. Analysis of postural differences between SR and WR sows at the same parity included standing (**A1**), ventral lying (**B1**), lateral lying (**C1**) and sitting (**D1**). Analysis of postural differences in different-parity sows in the same PLR group included standing (**A2**), ventral lying (**B2**), lateral lying (**C2**) and sitting(**D2**). * *p* < 0.05.

The frequency of the standing behavior of P0 sows with WR was significantly lower than SR (*p* < 0.05; Figure 1(A1)), and the frequency of the sitting behavior of sows with WR was significantly higher than SR (*p* < 0.05; Figure 1(D1)). The frequency of lateral lying behavior of P2 sows with WR was significantly lower than SR (*p* < 0.05; Figure 1(C1)), whereas the frequency of sitting was significantly higher in the WR group (*p* < 0.05; Figure 1(D1)). The frequency of ventral lying behavior in P5 sows with WR was significantly higher than SR (*p* < 0.05; Figure 1(B1)), and the frequency of lateral lying in sows with WR was significantly lower than SR (*p* < 0.05; Figure 1(C1)). Within each PLR group, the frequency of standing and lateral lying behaviors at P5 was significantly lower than at P0 (*p* < 0.05; Figure 1(A2,C2), respectively). The frequency of ventral lying and sitting behaviors increased with increasing parity (*p* < 0.05; Figure 1(B2,D2), respectively).

#### 3.1.2. Oral Behaviors of Confined Sows with Different Parity and Strong/Weak PLR

**Figure 2:**
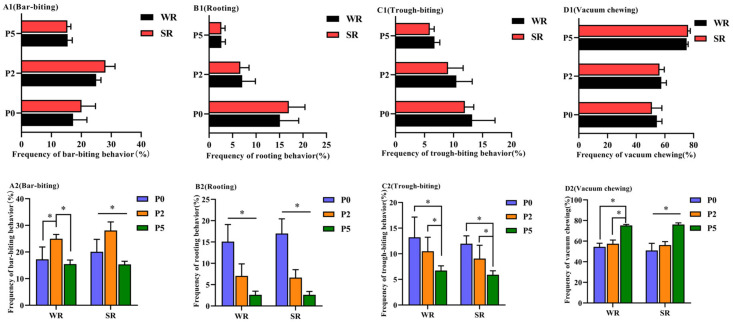
Oral behaviors of confined sows with different parity and strong/weak PLR. Analysis of differences in oral behaviors between SR and WR sows at the same parity included bar-biting (**A1**), rooting (**B1**), trough-biting (**C1**) and vacuum chewing (**D1**). Analysis of differences in oral behaviors in different-parity sows in the same PLR group included bar-biting (**A2**), rooting (**B2**), trough-biting (**C2**) and vacuum chewing (**D2**). * *p* < 0.05.

In sows of the same parity, no significant difference in the frequency of oral behaviors was observed between SR and WR sows (*p* > 0.05; Figure 2(A1,B1,C1 and D1), respectively). Within each PLR group, the frequency of bar-biting, rooting, and lateral lying behaviors was significantly lower at P5 than at P0 (*p* < 0.05; Figure 2(A2,B2 and C2), respectively), whereas the frequency of vacuum-chewing behavior was significantly higher at P5 than at P0 (*p* < 0.05; Figure 2(D2)).

### 3.2. Sucrose/Quinine Response

**Figure 3:**
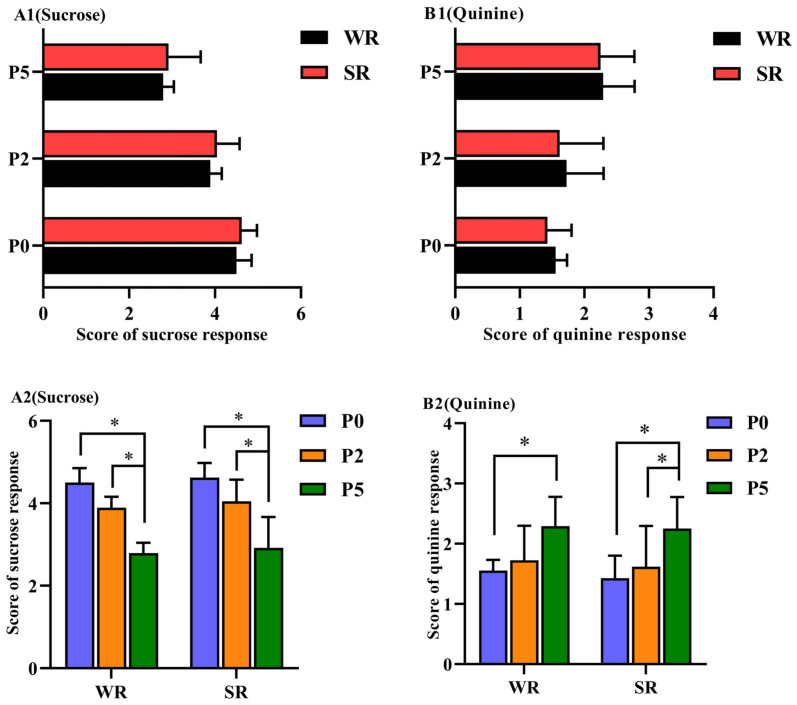
Sucrose/quinine response of confined sows with different parity and strong/weak PLR. Analysis of sucrose/quinine response score differences between SR and WR sows at the same parity included sucrose response score (**A1**) and quinine response score (**B1**). Analysis of differences in sucrose/quinine response score in different-parity sows in the same PLR group included sucrose response score (**A2**) and quinine response score (**B2**). * *p* < 0.05.

Regarding same-parity sows, no significant difference in the score for sucrose and quinine response was observed between SR and WR sows (*p* > 0.05; Figure 3(A1,B1), respectively). Within each PLR group, the sucrose response score was significantly lower at P5 than at P0 (*p* < 0.05; Figure 3(A2)), whereas the quinine response score was significantly higher at P5 than at P0 (*p* < 0.05; Figure 3(B2)).

### 3.3. Novel Object Test

**Figure 4:**
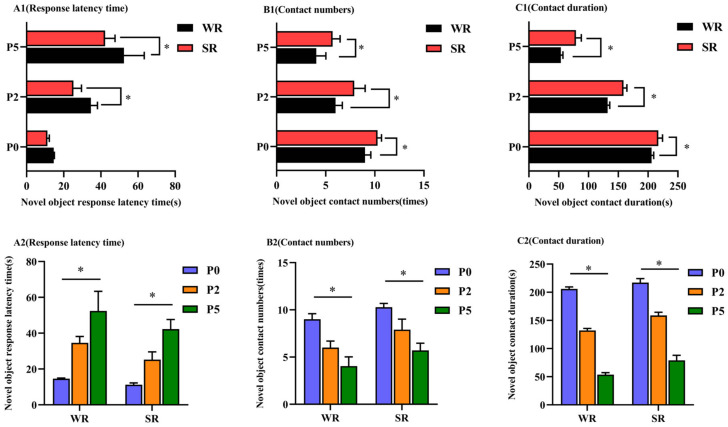
Novel object test of confined sows with different parity and strong/weak PLR. Analysis of novel object test differences between SR and WR sows at the same parity included response latency time (**A1**), contact numbers (**B1**) and contact duration (**C1**). Analysis of novel object test differences in different-parity sows in the same PLR group included response latency time (**A2**), contact numbers (**B2**) and contact duration (**C2**). * *p* < 0.05.

In the same parity, the response latency time of WR sows was significantly longer than SR sows (*p* < 0.05; Figure 4(A1)), whereas the number of novel object contacts and contact duration of WR sows were significantly lower than SR sows (*p* < 0.05; Figure 4(B1,C1), respectively). Within each PLR group, the response latency time gradually increased (*p* < 0.05; Figure 4(A2)), and contact numbers and contact duration significantly decreased with higher parity (*p* < 0.05; Figure 4(B2,C2), respectively).

## 4. Discussion

PLR is a part of visual function tests, and is used in clinical situations as an important indicator to evaluate factors such as potential pathologies of the central nervous system, the depth of anesthesia, and severity of certain diseases [38]. The PLR test is also a novel approach for the evaluation of neuropsychiatric disorders such as depression and anxiety [25]. From a psychophysiological perspective, pupil response is modulated by emotionally evocative stimuli [39]. Our results showed significant differences in behaviors and affective states, including postures and novel object contact, between WR and SR sows.

The behavioral expression of sows partly reflects their biological needs and adaptability to their environment. Stereotypic behaviors of pigs are adaptive responses to adverse environmental stimuli, and stereotypies of sows may include postural behaviors and oral behaviors such as ventral lying, lateral lying, sitting, rooting, trough-biting, and vacuum-chewing [40]. In human patients with depression and anxiety disorders, decreased sympathetic nerve function results in reduced physical activity [41], and patients with schizophrenia and depression show reduced activity and symptoms of negative volition, decreased motivation, and anhedonia [42]. The results of the present study suggested significant differences in behavior of same-parity sows in PLR groups, as the standing and lateral lying behaviors of WR sows were less frequent than SR sows, and the ventral lying and sitting behaviors of WR sows were more frequent than SR sows. Interestingly, our results showed that standing behavior in P2 sows was significantly more frequent than in P0 or P5 sows in the WR group, rather than in the SR group. In our behavioral observations, we found that with the increase in parity, sows showed a similar stutter-like behavior, but we did not accurately record it. Sows were able to adapt within each environment through behavioral mechanisms [43]. We believe that the change in sows’ standing behavior with the increase in parity is influenced by many factors, such as affective state, body weight, and movement space in the crates. We observed no significant differences in oral behaviors in different PLR groups at the same parity; however, bar-biting, rooting, and trough-biting behaviors in P5 sows were significantly lower than in P0 sows, whereas vacuum-chewing behavior was significantly higher in P5 sows than in P2 and P0. This indicates that long-term space restrictions in sows results in reduced activity and symptoms of negative volition, decreased motivation, and anhedonia. We previously found that compared with the SR group, the WR group showed lower 5-hydroxytryptamine levels and higher cortisol, interleukin-6, and beta-endorphin levels [19]. 5-hydroxytryptamine is an important neurotransmitter in depression, and haloperidol can inhibit dopamine receptors and enhance dopamine conversion in the brain [44]. 5-Hydroxytryptamine is an inhibitory neurotransmitter that affects the emotional state, and low levels can aggravate depression. Inducers or antagonists of serotonin can increase or decrease stereotypic behavior in animals [45]. We found that the changes in behavior in different PLR groups sows might be the manifestation of their psychological disorder [19].

The sucrose response test has been used to assess motivation, depression, anhedonia, and related affective states in rodents [46,47]. Low levels of aversion to foods such as quinine can imply a predisposition to certain psychological disorders [48]. The research results of Zhu et al. [49] showed that mice in the depressed group had a lower preference for sucrose than mice in the healthy control group. Chronic stress can lead to changes in the glutamate energy system and cause excessive release of glutamate, which is a potential mechanism to trigger depression [50]. Rats subjected to chronic stress of different duration can display depression-like behavior, and their preference for sucrose will also be reduced [51]. Sucrose solution can cause enjoyment in pigs, and a decrease in the response to sucrose indicates that sows have anhedonia. Chronic stress leads to neurobehavioral alterations [46]. We observed that the response to sucrose in WR sows was slightly lower than that of SR sows, even though there was no significant difference between the PLR groups. Olney et al. [52] found that mice subjected to long-term stress showed depression-like behavior, increased quinine consumption and quinine preference. Scinska et al. [53] found that the taste preference of mice in the depressed group changed and the highly-depressed group had a higher preference for quinine. We found that the response for quinine in WR sows was slightly higher than SR sows; even though there was no significant difference between the PLR groups. Therefore, we deem that WR sows may experience affective states such as depression and anhedonia. The response to sucrose was significantly lower and the response to quinine was significantly higher in sows of P5 than in sows of other parities, suggesting anhedonia in long-term space-restricted sows. In mice, the preference for sucrose in depressed individuals is significantly lower than normal mice [49]. Paul et al. [29] found that maternally-deprived rhesus monkeys showed reduced sucrose intake and increased quinine consumption than the control group. They suggested that maternally-deprived rhesus monkeys do not display gustatory signs of anhedonia, but rather of insensitivity to gustatory stimuli.

Animals in prolonged stressful environment develop a psychological response of fear. Fear is arguably the most commonly investigated emotion in domestic animals. The novel object test is a common type of fear test [54]. Our results revealed that the number of contacts and contact duration in WR sows were significantly lower, and the response latency time in WR sows was significantly higher than SR sows. This indicates that PLRs in confined sows are correlated with behavioral responses to novel objects, and WR sows may thus suffer from severe stress. Under the same work pressure, healthy people and depressed people appear in the same group [55]. A study used behavioral cognitive tests on major depressive disorder (MDD) patients. They found that MDD patients were more sluggish in responding to novel objects, showed a lack of focus, difficulty with divided attention, difficulty with decision making, difficulty thinking quickly, and difficulty learning new things [56]. Dickson et al. [57] found that depressed participants reported fewer approach goals and their reaction to novel objects took longer than control participants. This indicates that animals with depression will avoid the possibility of higher contact with novel objects. We suggest that the WR sows were more depressed than SR sows. With increasing confinement time, the number of novel object contacts and contact duration in P5 sows were significantly lower than in those of other parities, and the response latency time in P5 sows was significantly higher. These results suggest that an increase in confinement time may aggravate fear, reduce the number of novel object contacts numbers and duration, and delay the novel object latency time of sows. Stress responses are more severe, and exploratory behaviors as well as novel object contact numbers and duration decrease with increasing parity in sows [11]. In human patients with severe depression, novel object tests showed that most depressed patients were less responsive to novel objects [56,58]. Thus, novel object responses can be expected to be reduced in animals with depression, which is consistent with the results of our study.

## 5. Conclusions

There were significant differences in behavior and responses to novel objects between different WR and SR sows, and the affective state of sows changed significantly with increasing parity. The affective state of WR sows at P5 was consistent with depression. PLR may be a potent indicator for evaluating the affective state of sows. Our study may help establish a welfare evaluation system and enhance research on the welfare of sows.

## Figures and Tables

**Table 1:** Experimental sows grouping design.

Number of Sows	WR	SR
**P0**	*n* = 6	*n* = 7
**P2**	*n* = 6	*n* = 7
**P5**	*n* = 8	*n* = 8

Notes: P0 (parity 0): first gestation sows; P2 (parity 2): pregnant sows that have produced two litters; P5 (parity 5): pregnant sows that have produced five litters.

**Table 2:** Categories and definitions of postures.

Categories of Postures	Definition
standing	The stretched limbs maintain an upright posture of the body.
ventral lying	The chest and belly touch the ground, and the forelimbs extend forward or under the body.
lateral lying	The entire flank of the sow contacts with the ground, and all four limbs can be seen.
sitting	The buttocks of the hindlegs touch the ground, hind limbs are angled, and the forelimbs are upright to support the forebody weight.

**Table 3:** Categories and definitions of oral behaviors.

Categories of Oral Behaviors	Definition
bar-biting	Licking, nibbling, sniffing, and lifting the arched railings.
rooting	Close to or touching the ground with sniffing, licking, gnawing, or arching.
trough-biting	Licking, nibbling, sniffing, and gnawing the feeding trough.
vacuum-chewing	Chewing motion when there appears to be no food in the mouth.

**Table 4:** Scoring standards for the sucrose test.

Score	Definition
1	Sows avoid the source of the sucrose injection, their snout is close to the ground or moving back.
2	Sucrose is injected into the mouth of sows, but sows do not drink sucrose, and there are no marked or only minor changes of the snout.
3	Sow’s posture suggests it is drinking sucrose, and the snout shows obvious tension and closure of the jaw, but the frequency of tongue extension is low.
4	Sows show enjoyment by opening their mouths and sticking out their tongues, with a faster frequency of tongue extension and a larger range in tension and closure of the jaw.
5	Sows quickly stretch their tongues and approach the jet source, their tension and closure of the jaw are quickly with fluid sputtering, and the frequency of blinking is higher.

**Table 5:** Scoring standards for the quinine test.

Score	Definition
1	The jaw of sows is wide open and they rub their snout, accompanied by shaking the head and trampling on the forelimbs.
2	The opening and closing of the jaw of sows causes fluid to flow out of the mouth, alternately showing enjoyment or disgust behaviors.
3	Sows stick out their tongues and lick, the frequency of tension and closure of the jaw is higher.

**Table 6:** Categories and definitions of the novel object test.

Categories	Definition
Contact numbers	Number of times the sow sniffed, bit, or manipulated the novel object.
Response latency time	The time between the novel object being placed in the crate and first contact by the sow.
Contact duration	The time from the first contact with the novel object in the crate to the time when the sow leaves the novel object.
